# Proposal for the User-Centered Design Approach for Health Apps Based on Successful Experiences: Integrative Review

**DOI:** 10.2196/14376

**Published:** 2020-04-22

**Authors:** Guillermo Molina-Recio, Rafael Molina-Luque, Ana M. Jiménez-García, Pedro E Ventura-Puertos, Alberto Hernández-Reyes, Manuel Romero-Saldaña

**Affiliations:** 1 Department of Nursing University of Córdoba Córdoba Spain; 2 Department of Occupational Safety and Health Córdoba City Hall Córdoba Spain

**Keywords:** mHealth, user-centered development, focus groups, discussion groups, interdisciplinarity

## Abstract

**Background:**

Different strategies encompassed within mHealth have shown themselves to be effective for maintaining good health or controlling certain diseases. However, there is usually a very high rate of abandonment of health apps. Therefore, it would seem obvious that there is a need for involving the end users (whether they are health professionals, patients, or both) in the design process from the early stages in order to enable their needs and characteristics to be identified. In this sense, it is common knowledge that focusing on the user permits the consideration of valuable details aimed at making the correct adjustment between the patient, the technology, and the organization of attention.

**Objective:**

The goal of the research was to propose a methodology based on the review of previous successful user experiences in setting up health apps by using qualitative techniques (focus groups and discussion groups) that includes the participation of information technology and health professionals and the patients themselves.

**Methods:**

An integrative review was made of studies in which a qualitative methodology was employed mainly through focus and/or discussion groups for the design and development of health apps, consulting diverse databases (PubMed, Scopus, and Proquest) with the following search strategy: “mHealth AND apps AND focus group OR discussion group.” A total of 69 papers were included in the review.

**Results:**

A proposal structured in 4 sessions of variable duration was made in which information technology and health professionals and patients take part: composing, preparing, and organizing contents (session 1); testing structure and usability (session 2); does the app fit the needs of end users? (session 3); and last testing—keep on improving (session 4). Throughout the sessions, we propose studying aspects like previous user experiences in mHealth, barriers to the adoption of mHealth, interface contents, management and browsability, usability, perceived quality, security and privacy, capacity to self-manage disease with the app, ergonomics, and glanceability, etc. Specific tools that have proved useful in previous research for measuring these aspects are presented.

**Conclusions:**

These work sessions would be based on predominantly qualitative methodologies although, as they evolve, validated questionnaires permitting the assessment of the objectivity of certain technical aspects could be incorporated. With this proposal, a project centered on end users could be effected, responding to their needs. However, this requires validation that will be made via implementation in the development of health apps, with the subsequent measurement of results in terms of adherence and improvement in the clinical variables of the end users.

## Introduction

Mobile technology already forms part of our daily lives, and its presence is increasing rapidly. It is estimated that in 2019 there were over 2.7 billion smartphone users and around 1.4 billion tablet owners worldwide [1]. In addition, technical improvements in mobile devices, including larger screens, higher resolution, increase in browsing speeds, and development of many thousands of mobile apps with a multitude of new functionalities [2], have meant a genuine social and cultural revolution reaching all strata of society. As a result, the incorporation of mobile technology into our daily tasks has triggered changes in the way in which we live, work, communicate, and relate to each other socially [3].

According to the Global System Mobile Association, there are more devices connected to the network than people in the world. In 2017, 7.42 billion mobile connections were identified, whereas the population census in the world was of 7.23 billion [4]. Another relevant fact that helps to size up the magnitude of this technological trend is that, in 2014, for the first time, the number of accesses and the browsing time on the Web through mobile devices exceeded those made with desktop computers [3,5-7]—so much so that the future of technology and that of the mobile telephone are considered to be equal and it is very difficult to distinguish between one from the other. Thus, it is thought that within a few years, we shall be able to dispense with the adjective mobile when referring to technologies, as they will all have that characteristic [3].

The health care field has not been alien to this revolution. The term mHealth (mobile health) was used and defined for the first time in the year 2000 [8]. This concept was subsequently employed at the mHealth Summit 2010 of the Foundation for National Health Institutes to refer to “the rendering of medical attention services by means of mobile communication devices” [9], and nowadays this is understood globally as being medical practice and public health based on the use of mobile devices [10]. Since then and up to the present, around 40% of the more than 300,000 apps available in the different apps stores are related to health topics, with those focusing on disease monitoring and management standing out [11]. Different strategies included in mHealth, from simple phone calls or sending of texts (short message service, or SMS) to the use of apps as a support for clinical decision making or telemedicine, have shown themselves to be effective in the communication between patients and health professionals, in changes toward healthy lifestyles (giving up smoking or increasing physical activity), in the improvement of disease management (in diabetes or asthma, for example), and in the increase in adherence to treatments [12-15].

Further, it has been demonstrated that certain functionalities that are implicit in the habitual use of smartphones, like dissemination of information, possibility of self-monitoring with easy and intuitive record systems, interaction between users or using gamification strategies, also have positive effects on the state of the users’ health [16].

We must not forget that the popularity, mobility, and technical capacity of these devices mean that, as many people have them and are never separated from them, it is possible for synchronization between the health professionals and the patients without needing the former’s physical presence, either to give them special care or to warn them about any risks or changes in their health that require more urgent attention [15].

In this respect, a national survey of habitual users of mobile apps in the United States demonstrated that 58.23% of them had installed at least one health app, nutrition and physical activity ones being prominent. However, many had given up using them or had uninstalled them, mainly due to lack of time for entering the data; lack of interest; because after downloading the app for free, there were hidden costs that only appeared after a trial period (freemium models); difficulties in using them, or because of data being shared in the social networks or among groups of friends that they did not want to divulge [17].

In essence, and as reported in some other works [3,16,17], the abandonment rate with these apps is usually higher when a user has a bad experience. More than half of the customers who either uninstalled apps or did not have any continuity in their use claimed this reason, despite the apps being indispensable for their health care, especially in the case of chronic diseases. Therefore, as recommended by Alonso-Arévalo and Mirón-Canelo [3], any public or private entity involved in the design, development, and implementation of an app related to the health field should take into account all of these aspects and highlight their functionality, their being easy to use, their compatibility, performance, and safety.

In fact, it would be fitting to involve the end users of the apps (whether they be health professionals or patients, or both) in the process of designing them during the early stages in order to identify their needs and characteristics. In this regard, it is known that a user-centered approach permits the contemplation of useful details aimed at forging an adequate relationship between the patient, the technology, and health care organizations [18]. This participation of end users and health professionals in all stages of app development could result in an increase in their commitment and an improvement in integration, self-management, and health results, since most apps in which end users and health professionals did not participate in development have been seen to fail [19]. Besides, there is a consensus on the suitability of the use of qualitative techniques that permit the inclusion of all the actors implicated [20], in which the focus and discussion groups stand out [21]. However, although certain conceptual frameworks that could serve as a guide for setting up health apps have been proposed [22], there is no clear evidence-based proposal for the sequence and contents of each of the sessions.

The main objective of this study was, therefore, to suggest a methodology or guidelines (including tools for assessment) based on the review of previous successful user-centered experiences for the development of health apps by means of qualitative techniques (focus groups and discussion groups) that include the participation of information technology and health professionals and patients themselves.

## Methods

### Study Design

An integrative review was completed of studies in which a qualitative methodology was employed (ie, focus and/or discussion groups) for the design and development of health apps. The electronic databases consulted were PubMed, Scopus, and Proquest, with the terms “mHealth,” “apps,” “focus group,” and “discussion group” forming the search algorithm “mHealth AND apps AND focus group OR discussion group.” To locate papers not indexed in these databases, a manual search in JMIR journals (especially in e-collections) was performed. Analysis and selection of the manuscripts was performed by two experts in epidemiology and quantitative research (who assessed the scientific quality of the manuscripts) and one researcher with extensive experience in qualitative research (who assessed the quality of the information provided on the techniques used and the evolution of the sessions). The manuscripts were also evaluated by two researchers with previous experience in the development and evaluation of health apps.

### Eligibility Criteria

**Figure 1:**
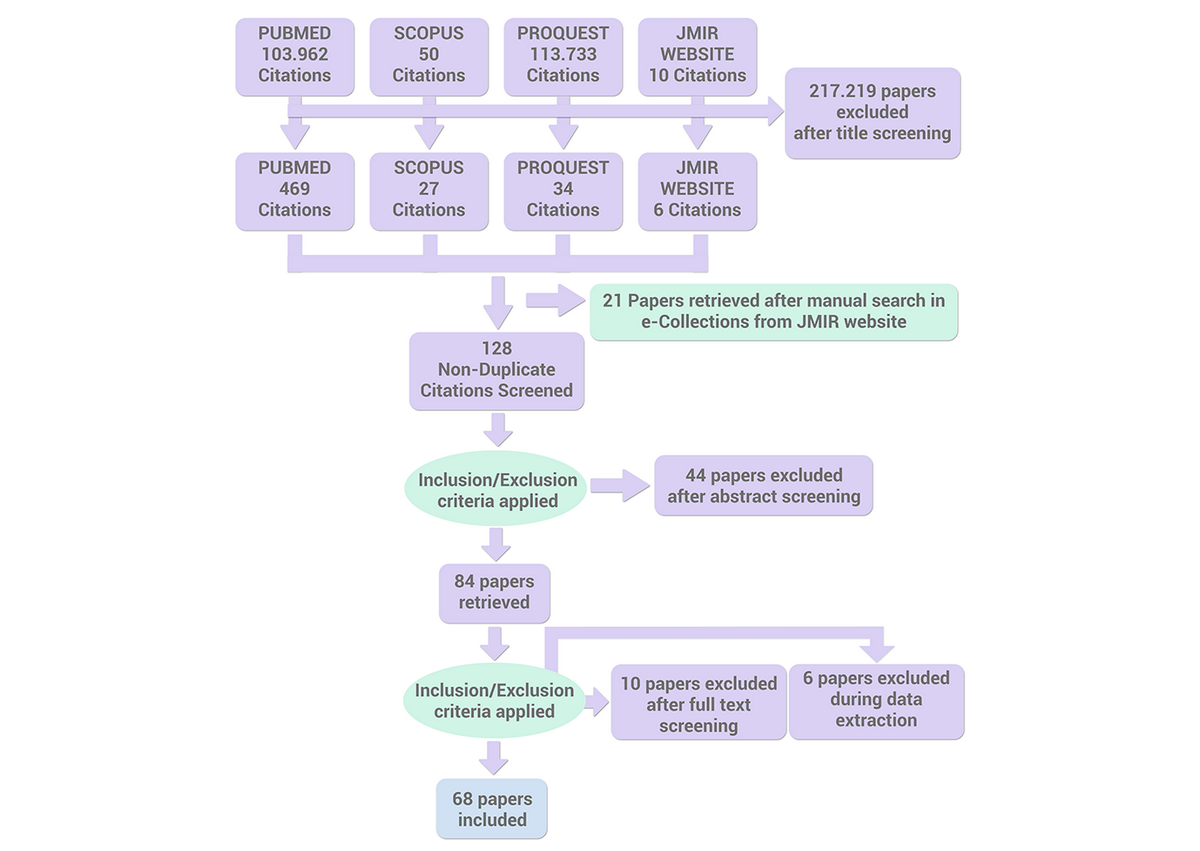
Paper selection flowchart.

The publications were located and selected between January 2000 and June 2018. Only those written in English or Spanish that were available as a complete text were considered. A reverse investigation was also made to prevent zones of silence. Articles referred to by the studies reviewed, which a priori would not have been found in the databases consulted, were chosen. Finally, 69 articles were included in the review (Figure 1).

The following information was extracted from the selected papers: type of study, number of participants, duration of the intervention, methodology used for the design of the apps, number of sessions held, main results, and questionnaires and other validated tools used in the sessions. If the manuscript included specific information on the questions used in the focus or discussion groups, it were recorded verbatim. With regard to the type of information used to make the proposal, the technique employed in developing the mobile app was followed: what type of information and how the latter was obtained for the apps design by means of the use of focus and/or discussion groups, number of participants recommended for training, duration of the sessions, how to evaluate the results obtained by the researchers, and, especially, which qualitative techniques contributed to the final state of the app.

This information was synthesized in order to establish a methodological proposal that included all sections considered for the design, development, and start-up of an app that attends to the needs of the end users and can serve as a guide for researchers and software developers with a view to offering products more in accord with the real demand on the apps health care market.

The decision on the type of session to be held for designing user-centered health apps (focus or discussion groups) and their duration was made following the recommendations of Lane et al [23], Sáez et al [24], and Savin-Bandenet al [25].

## Results

### Four Sessions for Implementing User-Centered Health App Design

Based on the articles reviewed, a method was proposed for the development of health care apps through qualitative techniques (focus groups and discussion groups) structured over 4 sessions of variable durations that included information technology and health professionals and patients. Diverse works with successful results have indicated that the fundamental objective of the inclusion of all these actors is to produce a design centered on end users that permits the detection of their needs, tests new behavior change concepts [18], increases their adherence to the developing app, and obtains positive behavior changes in health [26]. This practice permits one to unite the health care and information technology professionals’ technical knowledge, forming interdisciplinary teams that provide better results and a greater research impact [9], all of which are more positively valued by the users [27].

**Figure 2:**
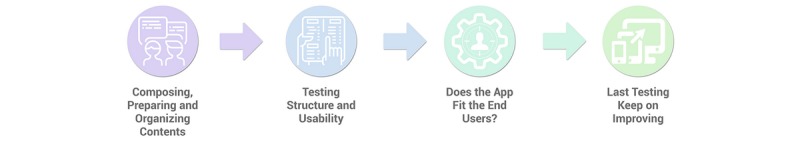
Steps in the design of user-centered health apps.

A basic outline of the structuring of the sessions and the fundamental topics dealt within them is shown in Figure 2. In Multimedia Appendix 1, readers can find verbatim quotations from participants (patients, stakeholders, or health care professionals) when qualitative tools (focus or discussion groups) were applied.

### First Session: Composing, Preparing, and Organizing Contents

#### Session Overview

Given the generalist characteristics of the introductory themes to be addressed in the first session and the importance of its being an open debate so that the largest amount of opinions and attitudes are aired about the development of a certain health app, a discussion group is proposed with health professionals, software developers, and patients or end users. This is considered to be more appropriate than a focus group because it encourages the participants to work together, thus obviating the influence of a moderator [23,24]. The discussion group will have approximately 10 participants, last between 60 and 90 minutes, and address topics on contents and mobile health.

#### Previous User Experiences in mHealth

**Figure 3:**
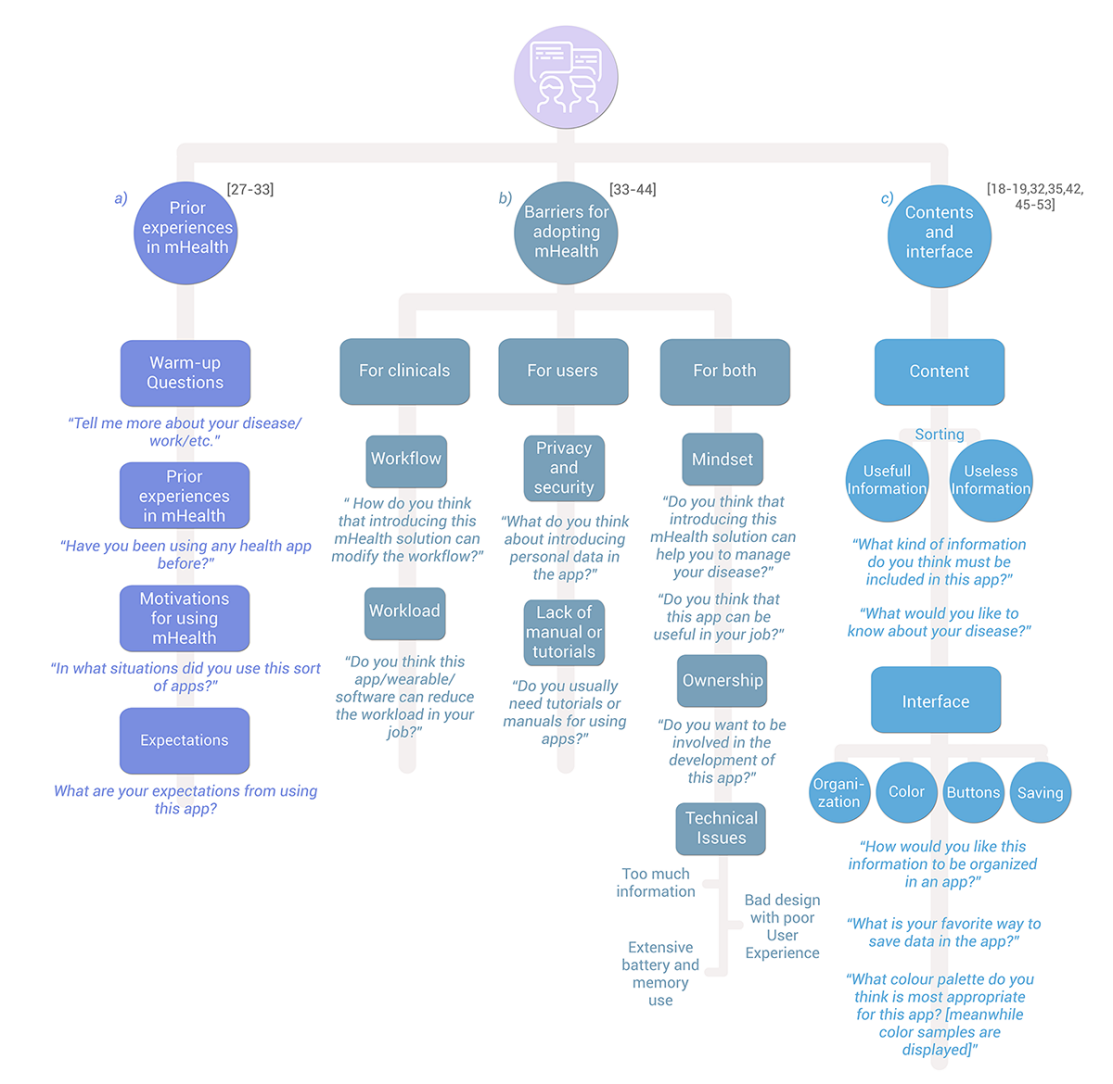
Session 1: Composing, preparing and organizing contents.

Previous works have obtained positive results by assessing the previous experiences of the targeted end users and showing how they can influence the use, browsing, and capacity to apply knowledge offered to health care management before beginning to elaborate a technological solution. For example, the work done by Greenfield et al [28] included the study of the previous experiences, motivations, and expectations for the use of wearables as a mechanism for promoting health in truck drivers. Other similar initiatives were put into practice by Pulman et al [27] for the development of mobile apps for adolescents with type 1 diabetes, Mirkovic et al [29] in the design and contents of an app for improving cancer patient care, and Cox et al [30] on the use of apps to enter anthropometric data or food intake. This strategy is very important in both elderly [31] and younger people [32]. In any case, it is important to include all final users, especially health care professionals and patients. In this regard, it is worth highlighting the experience of Lyles et al [33] in which a tablet app was designed for being used in the waiting room of a primary care consultation for complex patients that allowed prioritizing of the issues to be treated in these visits [33]. A proposal of the questions to ask, the topics to be addressed, and their sequence order is shown in Figure 3. See Multimedia Appendix 1 for quotes from participants in previous experiences.

#### Barriers to the Adoption of mHealth

Another, closely related aspect that can be addressed in this first session refers to the difficulties or impediments that end users may find in incorporating this type of technology into the health care field. Some user experiences have emphasized the need to collect this information in order to increase the effectiveness of the interventions based on mobile technology. In a pilot study performed by Hao et al [34], participants expressed their discontent with the reception of sample results in a laboratory via an SMS system. Their complaints arose from their lack of participation in the design, since they were only involved in it after its implementation; they expressed their wish to be informed early on about the project and wanted to have their opinions considered. This situation triggered a lack of motivation for performing that intervention and, more important, to give it continuity in time. Problems were observed deriving from the workload and flow and security and privacy of the data. Other studies highlighted more technical aspects as barriers, such as the lack of an appropriate instructions manual, too much information, an unattractive design, and excessive battery and internal memory consumption [33-41]. Last, it is commonly perceived that the patients do not really have any power to make decisions on their processes [41-44]. These perceptions can end up being obstacles (both for the professionals and the patients) that ought to be resolved starting from the first sessions. An extensive list of questions on obstacles and facilitator elements for the adoption of mHealth has been described in the research of Giunti et al [41]. A summary of the way in which to approach these issues in the first session is shown in Figure 3.

#### Contents and Interface

Without a doubt, one of the most important decisions in beginning to set up a health care app is organization of contents. Discussing these aspects with the end users is, therefore, fundamental, and there is ample evidence of its importance and positive effects. Shishido et al [45] developed a mobile app for compiling and reporting instructions for the evaluation of cardiometabolic risk that used graphic components like radio buttons and pull-down lists, giving rise to a standardization of frequently used data input to make it easy to complete forms. This proposal was made on the basis of suggestions from 5 nurses and a nutritionist, who gathered this type of information habitually in their work, and it was highly valued by all users. Casillas et al [46] also used qualitative methodologies through which they improved the contents and interface of a Web- and SMS-based system to guarantee access to comprehensive quality care for young adults surviving childhood cancer. Also, Kok et al [42], in their intervention on preventive treatment against the recurrence of calculi by means of a mobile app, reported that the use of striking colors and easy data input encouraged a greater adherence to the app. Many other research works have explored the contents and interface characteristics prior to the development of health apps for prevention, monitoring, or treatment of different diseases or creation of lifestyle changes: HIV [35], cancer and other chronic conditions [47], gout [48], multiple sclerosis [49], weight management [50], increasing physical activity [32], cardiovascular diseases [51], idiopathic arthritis [52] or chronic pain [53]. In view of these and other user experiences [18,19,26,33], some questions that could be included in this first session have been proposed and are shown in Figure 3. Some participant answers are also shown in Multimedia Appendix 1.

### Second Session: Testing Structure and Usability

#### Session Overview

After a first design and implementation of the app (that can be presented with a viable minimum product, mock-ups, or high- or low-fidelity wireframes), it is helpful to have a second session using focus groups so that the end users can give their opinions on the structuring of the information and the characteristics of its use. In this case, the group will put itself in the hands of the moderator as it needs to be directed in order to assess some specific aspects or to execute some tasks. The focus group will have approximately 10 participants, last between 60 and 90 minutes, and address topics on structure and usability.

#### Management and Browsing

**Figure 4:**
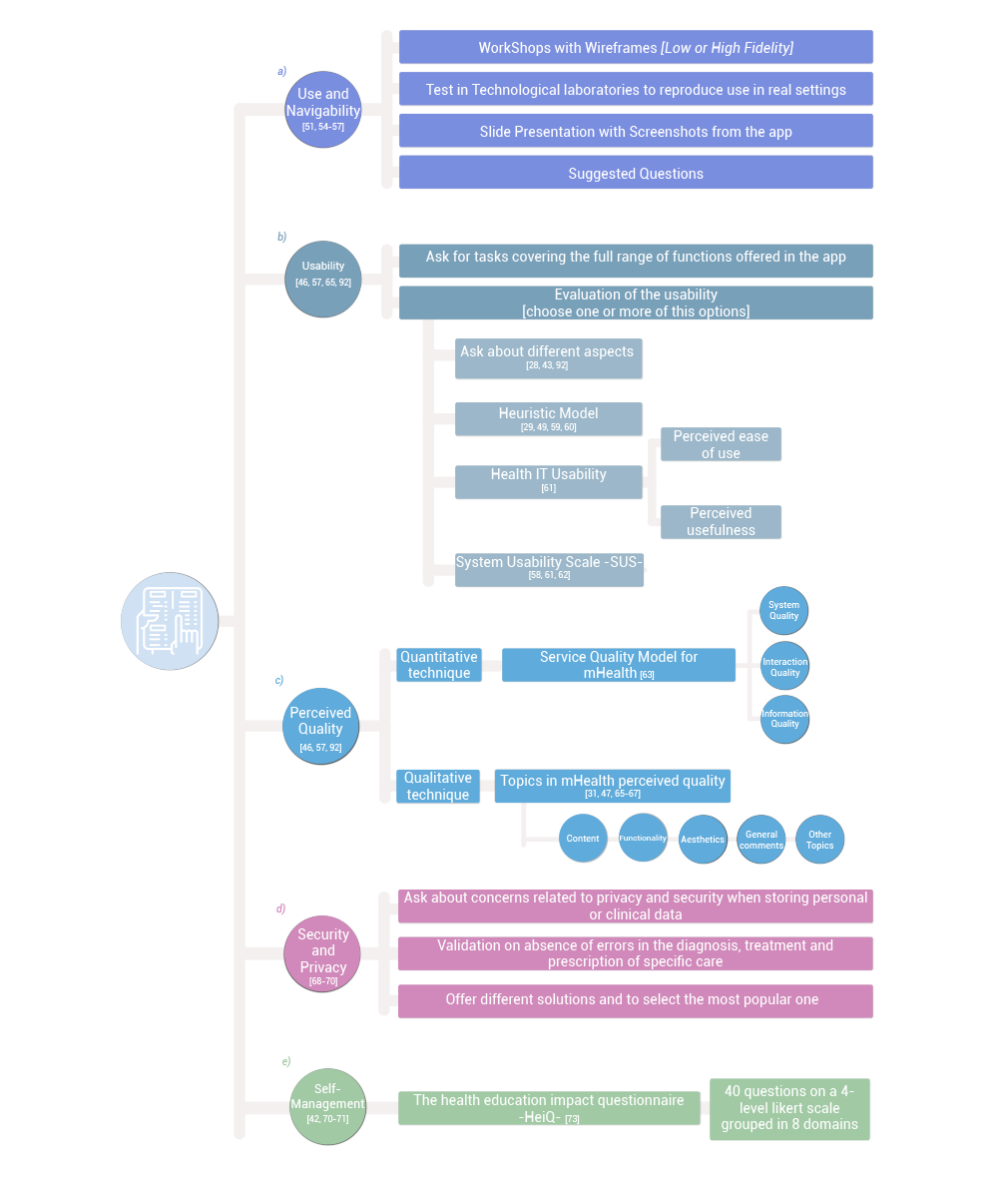
Session 2: Testing structure and usability.

The importance of easy management and navigation (browsability) in adherence to the use of health apps is evident [54]. The experience of Jakobsen et al [55] in the development of My Osteoporosis Journey, an app for the control of osteoporosis in recently diagnosed women, stands out. Different workshops were run in which wireframes were shown with different degrees of details (from low to high fidelity) and with a test in technological laboratories, where it was attempted to reproduce real conditions of use. Health professionals and women affected by the pathology reported a high degree of satisfaction with the app. In this same regard, some user experiences in the development of other apps for the self-management of illnesses or in their prevention have also resulted in products being well accepted by end users, when those aspects have been previously discussed with them [49,51,56,57]. A summary of the proposal for evaluating these aspects is shown in Figure 4 (more detailed information in Multimedia Appendix 2).

#### Usability

This factor represents possibly the greatest obstacle for the adoption of information and communication technologies in the health care settings. Generically, it refers to being easy to use, and its assessment is based on methods for identifying specific problems, centering on the user-app interaction and the study of the degree to which technology can be satisfactorily integrated into the task envisaged [46,49,51,58]. The latter requires device systems and characteristics to include facility of use, intuitive design and interoperability by means of esthetic issues [43], screen resolution, recharging time (for wearables), and the relevance of the data supplied [28]. It has been demonstrated that taking all of this into account in the early stages of development improves the predictability of the products and is time and cost saving [49].

Some specific methodologies that assess and improve the usability of a mobile app have been employed with good results in mHealth. For example, Mirkovic et al [29] used a heuristic usability model with high-fidelity prototypes in the design of an app to help cancer patients by contemplating the evaluation of 8 aspects that detected improvement areas [59]. This heuristic model, with small variations, was also used to assess the usability of apps developed for telerehabilitation [49] and increasing physical activity [60] of patients with multiple sclerosis. Likewise, Brown et al [61] created the Health Information Technology Usability Evaluation Model (Health-ITUEM) scale, which evaluates 9 aspects and gauges, basically, simplicity of use and perceived usefulness. These authors have also identified two possible reasons why the usability of the health apps has traditionally been reduced that should be addressed in group sessions: (1) small screen with low resolution and no keyboard or mouse available and (2) connectivity problems. Last, another option is the one used by Ribu et al [62], who applied an instrument developed in the 1990s for the self-management of diabetes using 10 Likert-type items, the System Usability Scale, to measure app usability [63]. This scale was also used by Vilardaga et al [58] to evaluate the usability of a mobile app to quit smoking in people with serious mental illness. These types of assessment (which are basically quantitative) can also be completed with a qualitative analysis through open-ended questions on the difficulty of performing different tasks assigned to the participants. A clear example of the use of this methodology is the research conducted by Mann et al [64] on the evaluation of the usability of an app aimed to improve iron intake and bioavailability in premenopausal women. Consult Figure 4 and Multimedia Appendix 2 for a summary of the way to explore usability in the design of health apps.

#### Perceived Quality

In a study by Akter et al [65], perceived quality is defined as the user impression of the excellence of the mHealth service. These authors developed an instrument to measure it in health apps with a scale comprising 22 Likert-type items grouped into 3 primary dimensions: system quality (user perception with respect to technical level of communication), interaction quality (between the service supplier and the user), and information quality (benefits of the service’s processes or what consumers receive as a result of their interaction with a supplier). These 3 dimensions comprise 8 other subdimensions (Multimedia Appendix 2). Other works based on the use of interactive wireframes and mock-ups [31,47,66,67] or apps already available in app stores [68] have measured quality by means of qualitative methods, asking about general aspects like contents, functionalities, or esthetics, leaving a margin for general comments or for any other item related to mHealth. Both approaches have been seen to be adequate for the measurement of perceived quality.

#### Security and Privacy

The inclusion of clinical experts in app design and considering the opinions of end users increases the security of the system objectively and ensures that the security is perceived, which increases use adherence. For example, Hilliard et al [19] involved patients with cystic fibrosis in the development of their app using semistructured in depth interviews and discussing design problems, among them those relating to security, and disclosed that most people’s concerns revolved around the storage of their clinical and personal data. Offering different alternatives (customization of the mode and type of data stored, inclusion of privacy options, etc) and listening to participant opinions permitted the development of an app that was perceived as being safer. Privacy has also been successfully explored in the development of apps for reminders of taking medication in HIV patients [69] and for stress management in cancer survivors [67], showing how for the former it is a fundamental aspect (“The ‘Did you take your medicine notification?’ is a problem. Did you take your medication? Anybody in their right mind is going be, ‘What do you mean you take medication?’ It lets them know you’re sick. Be hiding it from your family”) while for the latter is an unimportant topic (“My life is not that exciting” and “I have nothing to hide”). In addition, security in mHealth is also related to the diminution of errors in the transmission of information and the advice or health care given, so that, if necessary, this aspect should also be evaluated. Research projects like the ones conducted by the team of Surka et al [69] to improve the detection of cardiovascular diseases, Holmen et al [70] focused on the management of type 2 diabetes, and Jibb et al [71] centered on developing an app for treating pain in adolescents with cancer all constitute good examples of how to address these important aspects. Except for some cases [67], there is general agreement from all end users on the importance of the use of passwords to regulate access to their data [28,72].

#### Self-Management

One of the desirable characteristics offered by health apps (especially those directed toward controlling chronic diseases) caters to the possibility of empowering users to make decisions on their process (coping skills, target setting, self-monitoring, environment modification, etc) instead of merely providing the care prescribed by the experts [42]. Thus, in this second session, it is necessary to estimate the characteristics and functionalities the app should have to facilitate the self-management of one disease in particular [71]. Holmen et al [70] designed a low-intensity self-management intervention for patients with type 2 diabetes using a mobile app (Few Touch) and successfully used the Health Education Impact Questionnaire [73] to measure the impact of the app on the self-management of this disease. This questionnaire comprises 40 Likert-type items with 4 response levels grouped into 8 domains (Multimedia Appendix 2), and it could be employed in this second session to estimate the effect of the app and monitor its development, although it would be of interest to collect these data again after prolonged use of the definitive version.

### Third Session: Does the App Fit the Needs of End Users?

#### Session Overview

This will be completed several weeks after access to the version to be assessed so that end users can make an adequate evaluation of the app. This time period will vary according to the number of functionalities included in the app and its estimated daily use time and should be decided by the participants in the previous session. A focus group is recommended, with an estimated duration of 90 minutes and a similar number of attendees. Many of the topics to be addressed in this session are closely related to usability (or they have been seen in the previous session as part of it), but once the app’s development status has advanced, some of its characteristics should be revised individually. This would allow us to make a more exhaustive study of the different functionalities and the way to present them in the interface. It is recommended to gather information on the needs and desires of the user.

#### Acceptability

**Figure 5:**
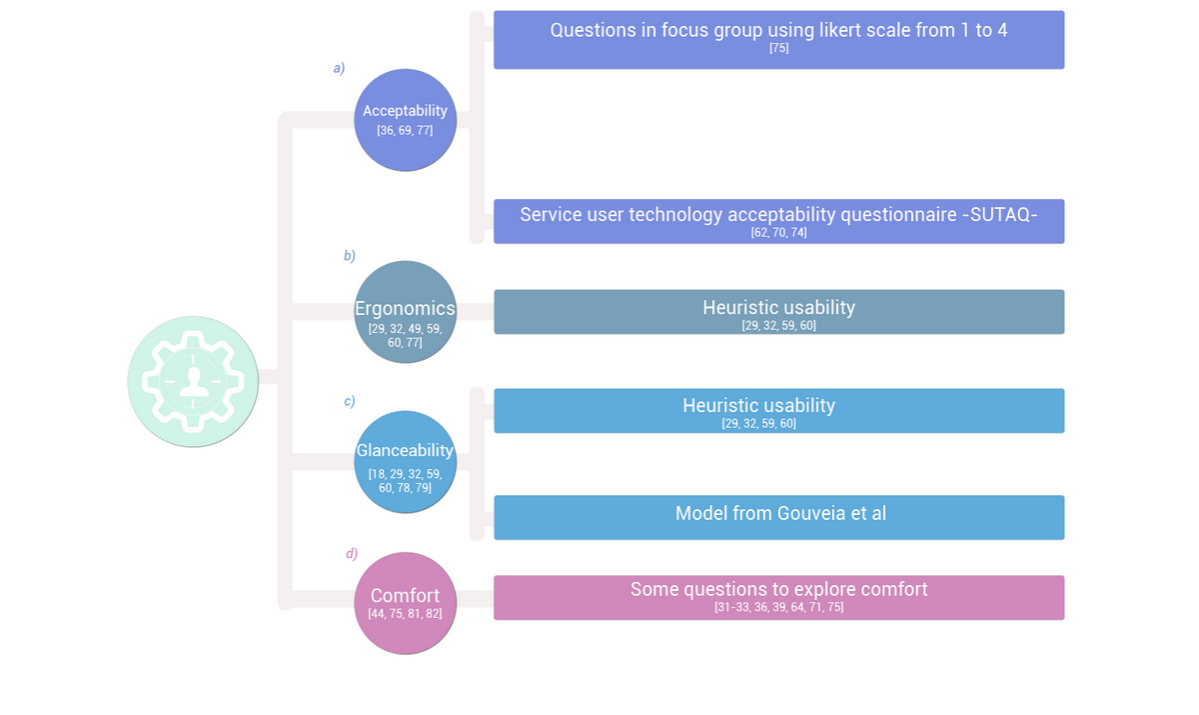
Session 3: Does the app fit the needs of final users?

Acceptability is an intimate concept directly related to usability, and it refers to the extent to which the patients are satisfied with a service and are willing to use it [62]. In the low-intensity intervention for type 2 diabetes patients of Ribu et al [62,70], the Service User Technology Acceptability Questionnaire was employed at a pilot phase with good results. This questionnaire contains 22 questions (that can be applied to satisfaction with an app or with any other technological solution) in the following domains: enhanced care, increased accessibility, privacy and discomfort, personal care concerns, kit as substitution (when the mHealth solution includes some device or wearable kit), and satisfaction [74]. Another alternative for the study of acceptability was demonstrated by Eisenhauer et al [75] in which, during a focus group, they used surveys based on a Likert-type scale and administered by the researcher to evaluate the satisfaction and use guidelines of an app for the self-monitoring of food and physical activity patterns. Also using focus groups, Dworkin et al [69] evaluated the acceptability of an app that showed an avatar for reminding HIV-positive men who have sex with men to take antiretroviral medication (“He looks so real, and he’s a nice attractive man, and I’m going to ask him a lot of questions about medication! This is genius idea!”), while Duff et al [36] measured the degree of acceptance of the Medfit app for the improvement of cardiovascular disease self-management through changes in lifestyles (“I found the progress part very useful. I got a reality check when I saw what I was doing and thought I was more active than I am”). Similarly, Van der Weegen et al [18] investigated the requirements of users of a mobile device for stimulating physical activity in primary care, including the end users in the design process from an early stage. These authors demonstrated that centering on the users improves the relationship between them, the technology, and health care organizations, and, therefore, the acceptability of the tool. Other researchers expressed the same perspective when studying the health of war veterans [66], people wishing to lose weight [76], and those wanting to give up smoking [77]. Also, as Mirkovic et al [29] concluded, the acceptability of an app is influenced by the phase of the disease, since needs change and they influence the perception of the utility and acceptability of the properties of the system. Therefore, using different qualitative techniques, the characteristics of the app must be defined in chronic pathologies that include a progressive deterioration [29,36,69], and this could be relevant in this session or, in the case of this evolution being expected habitually, in earlier phases when interface and content aspects are addressed (first session). More details about tools to be used for evaluation of acceptability of a health app and some answers from focus group participants are shown in Figure 5 (with more detailed information in Multimedia Appendix 3) and Multimedia Appendix 1, respectively.

#### Ergonomics

With this characteristic, the extent to which the mobile app adapts itself to the needs of a patient is evaluated. One way of doing so is by means of the heuristic usability questionnaire [29,49,59,60], already mentioned, specifically in its fourth dimension, which refers to using with parsimony the resources available on the principal screens and of the state of the app [77] and limiting the use of pop-ups and other notifications [29]. Ergonomics ensures clarity and simplicity of use, so it can also be assessed through qualitative techniques such as focus groups. For example, Simon et al [32] showed that clarity and ease of use was well valued by users of an app promoting an active lifestyle (“I think the app is easy and very clear. And there is not too much in it. With some apps you are like ‘Where do I find that again?’ but that is not a problem with this one”). At this point, the work of the graphic designer and software developers should be centered on guaranteeing the adaptability of the app to different mobile devices (responsive features) and to the special needs of some end users.

#### Glanceability

This characteristic of mobile apps refers to the information being comprehensible at first sight or with occasional glances, requiring a minimum of attention and effort to understand it [78]. That is to say, this refers to the perception and interpretation of the information after users have given their attention to the interface, which can be measured on the basis of the speed and ease with which the messages from the app can transmit the information after being seen [79]. For instance, the inclusion of demonstration videos, images, and other audiovisual items and the use of widgets and showing results in graphics can increase it [18]. It can also be evaluated by the heuristic usability questionnaire [29,49,59,60], so its evaluation would be simultaneous to that of ergonomy. In addition, Gouveia et al [80] created a system with 6 glanceability aspects used to select the best of 21 physical activity trackers, and we believe this system could also be employed in making health apps. The 6 aspects proposed by these authors are as follows:

- Data summary (abstract): measure in which the data shown appear as being already processed and permit users to process, quickly become aware of, and reflect on their health behaviors
- Integration with existing activities: the degree to which the most relevant information for the user and the app is integrated into places frequently accessed and, therefore, commonly seen by users
- Comparison to target and norms: this aspect is employed to assess whether the app provides comments on the user’s progress in such a way that they are easily processed and evaluated by the users themselves, providing clear feedback
- Being actionable: another desirable quality as a part of glanceability is that the interface would offer effective feedback and information but also trigger short actions to fulfill the health goals proposed
- Leading checking habits: this refers to the results being presented not only as being user-friendly but that they should urge the users into acquiring the habit of verifying them (ie, systematically examining the screen of the app) and consulting their progress. For that reason, it is important that this information can be checked at a single, quick glance [18]. Novelty (that the app continually presents different types of data) and scarcity (when the behavior feedback is shown for only a limited time) are strategies which have proved to be effective for this purpose
- Proxy to further engagements: it is known that, with the passing of time, users stop consulting their data. An app with a high degree of glanceability will trigger moments not expected by the user that would act as signals and increase their commitment with the use and consultation of the data that the app offers. One strategy could be to present information that generates questions instead of giving replies; another could offer ideas that surprise the user

See Figure 5 (with more detailed information in Multimedia Appendix 3) for a summary of the tools shown to be effective for evaluating glanceability.

#### Comfort

With this term we refer to the capacity of the health app to make disease management easier, due to the employment of mobile technology assistance in the collection, processing, and analysis of health information [44,75,81]. It also means that the app under development, if it is for professionals, will be a more efficient and faster method for disseminating knowledge within the scientific community [37,45]. In this regard, Zanetti [82] recognizes the need to find strategies that strengthen scientific creativity, with research based on the setting up of new technologies. This aspect can be assessed in a focus group to define the characteristics the app should have to make management of disease easier [31-33,36,58,64]. For example, in the development and evaluation of an app for diabetic foot care [31], it was found that the content of the interface was too small for these types of patient, who usually also have retinopathy (“...that’s too small an interface for my eyes because I’ve had retinopathy, I’ve had laser surgery on both eyes, I’ve had cataracts removed off both eyes”). Given that there are no objective tools for evaluating comfort quantitatively in the use of a health app, this theme can be addressed in this session once aspects like the interface and usability, to which it is directly related, have been defined. Some questions used in prior studies [39,71,75] that evaluated this characteristic can be posed in this session and are found in Figure 5 (and Multimedia Appendix 3). See, also, some answers from participants in Multimedia Appendix 1.

### Fourth Session: Last Testing. Keep on Improving

#### Session Overview

The fourth and last session (with a similar duration and number of participants) should be held 3 weeks after the third session so that end users will have had time to test the last versions of the app. The fundamental goal of the session will be to define all characteristics and improvements and future development of the app, with the aim of obtaining a product that could be validated in real settings and with a larger number of patients. During this time, based on the user experience, participants will be able to reflect on the topics addressed previously and discuss the future of the app.

#### Proposals for Improvement

The aim of this block will be to explore whether any difficulties have appeared or any individual needs have been detected and assess the response offered by the app. In this way, the possibility of introducing improvements that allow us to give specific answers to the largest possible group of users will be considered.

As various works have shown, customization is important to users to satisfy individual preferences and disease management goals [19,56,80,83,84]. Similarly, a more formal or clinical language should be incorporated for some functions (description of pathologies) but more informal language for others (evaluation of conducts) if the users wish. As some authors have pointed out [42,72], it is important to strike a balance between the mobile app being attractive and amusing but not so much so that it discredits the sense of authority. In this sense, Mirkovic et al [29], using the heuristic usability model [49,59,60] during the development of an app for assistance in the management of their illness to cancer patients, found that the users demanded the possibility of having a configuration menu to select the visibility of the principal functions. The work of Koskinen and Salminen [85] also stands out, in which the elaboration of an app for increasing healthy living habits highlighted the importance of the configuration of menus to augment use adherence. Some of the parameters recommended (some for new users and others for advanced ones) were to (1) enable or disable health parameters, (2) aggregate new properties to an existing parameter, (3) modify the presentation of data, (4) add new parameters, (5) change parameters and existing properties, and (6) aggregate or modify units (only through the XML edition). Open-ended questions in focus groups have also proved useful in assessing the need for customization for end users. Thus, for example, in the development of the app for taking antiretroviral medication discussed above, one of the participants commented on the need to customize the alarms and physical characteristics of the avatar [69]. Also interesting is the contribution of some users who participated in the design and evaluation of an app to facilitate self-monitoring and management of mood symptoms in young people, by revealing that it could be important to let each person choose colors to better define the moods which participants could experience [86]. Other works also valued positively personalization for elaborating contents that could be sent by mail to the doctor [48], further adapting the contents of the messages according to the achievements recorded [87], etc.

People’s needs change throughout the health-disease process in which they are immersed. For that reason, a health app, especially for end users who have chronic conditions, should be capable of accommodating their preferences and objectives in managing their illness in terms of the stage at which they find themselves [19,43,88,89]. Flexibility has been successfully measured as part of the study of heuristic usability (component 6) [29,49,59,60] and of the model Health-ITUEM [61].

#### Usefulness

This characteristic can be assessed by surveys (original and/or specific, for evaluating aspects closely related to the health theme addressed or validated and focused on improvement in the quality of life, adherence to treatment, or advances in clinical parameters) [29,90]. Besides, there is sound evidence that the utility perceived in health apps maintains a direct relationship with the continuity in their use [91]. In any case, this should refer to the usefulness to illness management of monitoring and accessing information sources [19,34,54,72]. The usability study model Health-ITUEM [61] includes assessment of utility perceived as part of user satisfaction with the app; estimation of how easy it is to learn to use; perception of the skill needed to perform tasks (the extent to which the users trust their ability to do tasks using the system); speed of task completion; and flexibility or capacity to personalize the app. The usefulness of the developed apps can also be evaluated through specific questions in the focus groups [31,32,36,64]. For example, an app for the control of gout was considered helpful because it allowed the patient to become aware of what food could cause the attacks (“...so as I’m putting in an attack I can access the relevant triggers that have caused me issues in the past”) [48]. Another that offered telerehabilitation in patients with multiple sclerosis was considered suitable for presenting videos with different types of exercises [49] (“I think the exercise videos are good because a lot of the movements are what you do in therapy. So, this is along that line to get you moving more”), while users of another app to increase physical activity in patients with type 2 diabetes mellitus assessed the app’s ability to motivate (“It made me feel motivated.... I would (exercise) because I was afraid they were going to say, ‘Hey! Get off that sofa!’”) [87].

#### Hardware Limitations

One aspect of reality makes it difficult to develop health apps. Technology advances rapidly but the test equipment for the end user and software move slowly [59]. For example, one problem that has been highlighted in previous user experiences occurs during an attempt to migrate a Web-based system to a mobile platform (a fairly frequent practice in mHealth), a challenge for software developers and designers due to the limitation in screen size and input capacity of some devices [29]. General measures toward solving hardware limitations that have proved effective and whose application can be agreed upon in this session include the following:

- Provide support only to a limited number of functions in order to eliminate the variety of options not fundamental to mobile use [46]
- Show limited contents to reduce word count and facilitate better visibility and glanceability [85]
- Improve the interface with elements that permit easy data input [40,60]

**Figure 6:**
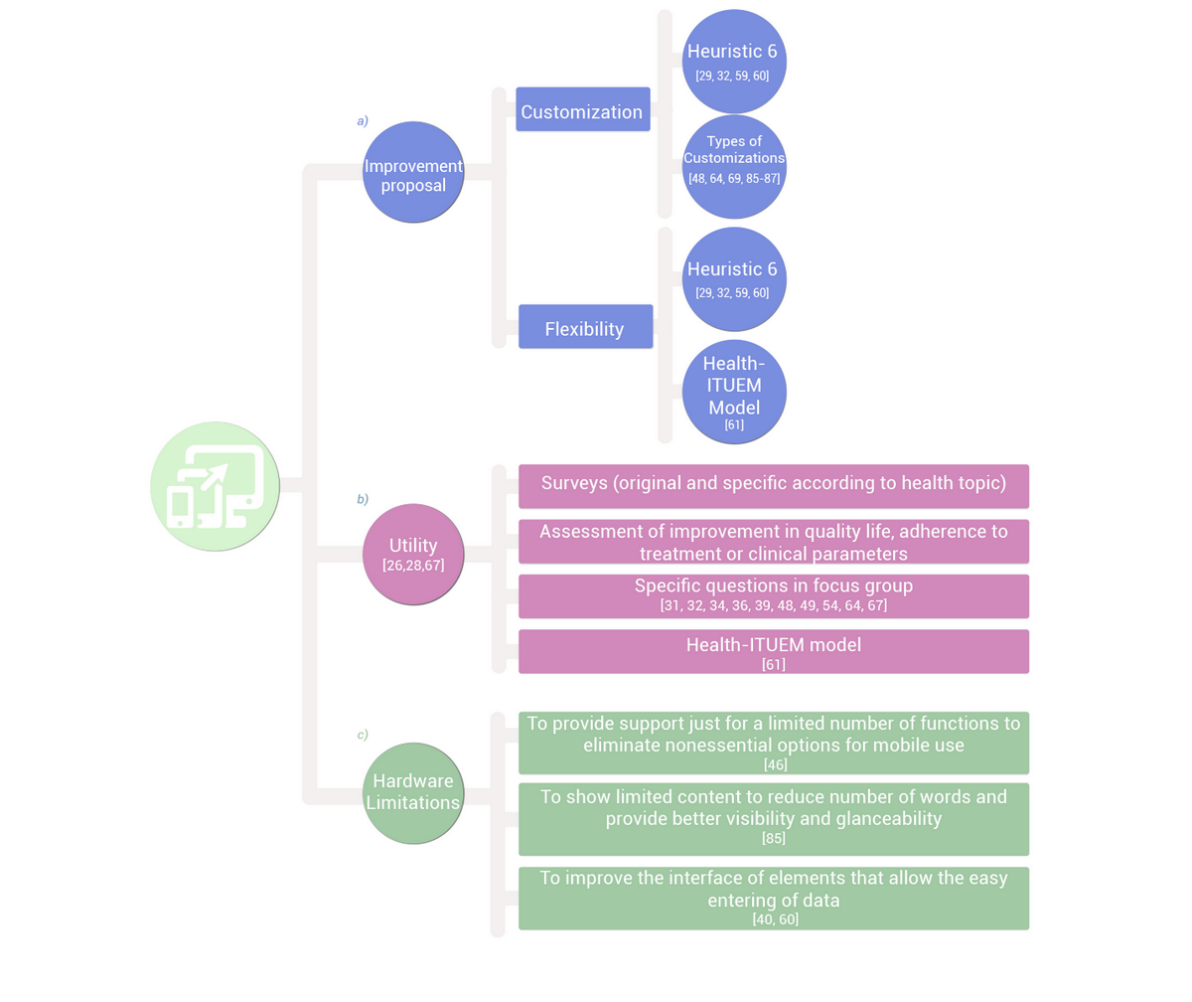
Session 4: Last testing. Keep on improving.

In view of the characteristics of the topics to be dealt with in this session and the continuity it maintains with the previous one, the focus group is also considered as being the most suitable qualitative technique, with a similar duration to the previous ones [25]. It is recommended that the same participants as before participate in it. A structured summary of this fourth session can be seen in Figure 6 (with more detailed information in Multimedia Appendix 4) while verbatim quotations from participants in the focus group are shown in Multimedia Appendix 1.

## Discussion

### Principal Findings

This is a proposal to implement the design and development of health apps using a user-centered approach. For this purpose and based on previous experiences of other researchers who obtained positive results, a sequence of 4 group sessions has been proposed. Scientific evidence has demonstrated the effectiveness of exploring in these sessions the needs, previous experiences, and difficulties experienced by the users of health apps, as well as improvements in usefulness and usability. These and other aspects will be essential to achieve adherence to healthy lifestyles or the adequate management of chronic health conditions.

### Limitations

The principal limitation of this work is that, despite the proposal being based on evidence brought by previous successful user experiences in the field of mHealth, the method proposed requires a specific validation to determine its efficacy. However, we have attempted to offer a logical sequence with specific tools and methodologies for each aspect to be evaluated in order to be flexible and open to improvements that permit the creation of a valid development framework for setting up health apps. Although this proposal has managed to encompass the best and most recent scientific evidence available in the mHealth field, other propositions including different tools or sequences could also be valid.

Other limitations are directly linked to the fact that we based the development of a health app on qualitative techniques. As other works have implied [29], by proposing to work with a reduced group of end users, it is necessary to consider that these users may not be an adequate representation of the target population. However, the size of the sample is more or less consistent with the general recommendations that have established that most usability problems can be identified with a smaller number of participants.

In addition, although qualitative methods have habitually used compilation of data in the development and validation of technology-based solutions, there are certain demands in data analysis that cannot be dealt with from this perspective: identification of the analysis unit, elaboration of constructs, mitigation of the effect of the dynamics within the group, variations between groups, inconsistency of data, etc. To alleviate these problems, the research team should train the focus group leaders in moderation techniques in order to enable them to focus the sessions on the topics related to the issue being addressed in the research [25,43]. However, employing validated questionnaires to measure certain aspects linked to the efficacy of the app developed could also remedy this lack.

As others have pointed out [92], these techniques can be subject to group biases. Nevertheless, they generate a natural open discussion, providing fruitful feedback on usability. For that reason, a design is proposed in which the participants are offered different iterations that are focused on concrete functionalities during the sessions. In any case, this is a natural consequence of agile iterative development.

It is also important to mention that the qualitative sessions take place in meeting rooms behind closed doors and not in real settings, which might modify the behavior of some of the participants. For that reason, as already mentioned, we recommend holding group sessions only once with each participant (except between the third and fourth session so improvements of the app versions can be evaluated). Also, the data collection techniques should only include fieldwork observations, follow-up surveys, and information sessions with moderators [25].

Finally, we would like to emphasize that this research does not aim to evaluate the effectiveness of apps, the methodology of their development, or the practical application. It would be difficult to achieve that objective, given that there is no protocol, proposal, or guideline that can be taken as a reference for the evaluation of the methodologies developed in the reviewed papers. For this reason, this manuscript aimed to provide a structured, user-centered scheme for health app design and development with effectiveness assessed in subsequent mHealth investigations. Nor did we try to identify which user experiences are vital and necessary to the effectiveness of the app itself (which could differ according to population, intervention, or pathology). For this reason, we have proposed a methodology in which these vital elements could be explored.

Despite its limitations, as reported by Peng et al [92], this type of research adds important qualitative evidence in the setting up of mHealth, since it permits access to important information for researchers and app designers, making development of an app with potential for the adoption of healthy lifestyles and improvements in self-care more likely.

### Conclusion

This work has proposed a 4-session methodology for the development of health apps, in which aspects such as the difficulties in adopting behavior based on mHealth, acceptability, browsability (ie, the ability to easily browse or navigate through the information offered by the app), usability, and interface study could be studied. These work sessions would be based on predominantly qualitative methodologies (focus and discussion groups); although, in their elaboration, they include validated questionnaires that permit the assessment of objectivity of certain technical aspects. There would be around 10 participants in the groups, with information technology, graphic design, and health care professionals and patients represented. Prior evidence tells us that, in this way, the app’s design will be focused on end users, attracting and responding to their needs and, therefore, increasing their adherence to using the app. This would result in positive changes in their attitude toward their health and an increase in their commitment and self-management of the health-disease processes. This proposal requires validation with subsequent measurement of results in terms of adherence and improvement in the clinical variables of the end users, either professionals or patients.

## Appendix

Multimedia Appendix 1 Sessions and themes with associated quotes.

Multimedia Appendix 2 Session 2: Testing structure and usability.

Multimedia Appendix 3 Session 3: Does the app fit the needs of final users?

Multimedia Appendix 4 Session 4: Last testing. Keep on improving.

## Abbreviations

Health-ITUEM: Health Information Technology Usability Evaluation Model
mHealth: mobile health
SMS: short message service
