# Supplementary material for: Proposal for the User-Centered Design Approach for Health Apps Based on Successful Experiences: Integrative Review
Source: JMIR Mhealth Uhealth. 2020 Apr 22;8(4):e14376. doi: 10.2196/14376 (PMC7203616; doi:10.2196/14376)
Supplement: Multimedia Appendix 1 [file mhealth_v8i4e14376_app1.docx]

| **Multimedia Appendix 1. SESSIONS AND THEMES WITH ASSOCIATED QUOTES** | | |
| --- | --- | --- |
| **Session** | **Themes** | **Verbatim lines (only when qualitative tool was applied) from participants (patients, health care professionals or other stakeholders)**  [In brackets number of reference] |
| **1A** | Previous experiences | *“I just haven’t bothered with any of it; it’s my age, I just don’t” [31]*  *“I finally bought an iPad not too long ago and I still don’t even know how to use that. I don’t know much about computers.” [33]* |
|  |  |  |
|  | Expectation | *“...a smartphone is way easier than a computer. You always have it by your side and you don’t have to wait until it’s ready to be used.” [32]* |
| **1B** | Workload | *“I’m interested in how this [the mHealth platform] would work around our end and to see how much...drudgery can be taken out of the work...‘cos...there's a lot of work processing records.” [35]* |
|  | Barriers (Privacy) | *“Even though it has codes and all kind of stuff…this app is online…Anybody can hack my email…I don’t want it to maybe one day, come out…if you say, ‘This app is here you can download it,’I will say:’No, thank you’.” [35]* |
|  | Barriers | *“I would love to do this because I know it helps your balance… I think the exercise videos are good, because a lot of the movements are what you do in therapy. So, this is along that line to get you moving more” [36]* |
|  | Mindset | *“I want to note the dates of when I have symptoms before I forget them, so that I can show the doctors, and help them better understand my illness and what is really going on.” [52]* |
|  | Privacy | *“Arthritis is quite personal so some things you might not want to share with other people.” [52]* |
| **1C** | Information adequacy | *“I would find it interesting to know how active I need to be to be healthy. But I need encouragement. And people need guidelines and ideas on how to be active.” [32]* |
|  |  | *“It’s [the written information] good because they’re apt sentences but they’re not lengthy ones, and I reading an app or data...it’s a real turn-off to read an inch or a long, long paragraph — so, short and sweet sentences are great.” [48]* |
|  | Content | *“Maybe food recipes is a big one, because we can eat healthy all we want but if we continue to eat the same grilled chicken breast, it will get boring. So maybe it could update all the time with different food recipes.” [50]* |
|  |  | *“The app should have information on arthritis…also news about research, involvement, and being able to talk to other people.” [52]* |
|  | Interface | *“I need to have something visual. I’m not a hearing learner. I’m a visual person.” [33]* |
| **Session** | **Themes** | **Verbatim lines (only when qualitative tool was applied) from participants (patients, health care professionals or other stakeholders)**  [In brackets number of reference] |
| **2B** | Usability | *“I like the way the sections are organized, and how it is not too overwhelming, but how I do have the option of finding out more if I want to.” [52]* |
|  |  | *“Well I had some issues when logging in, at first it took me about ten attempts to log in, but now it’s kept me logged in and that’s fine.” [65]* |
| **2C** | Perceived quality | *“I think it would cause us to get a good picture, get a fast picture, evaluate that with the patient so we don’t walk into an assumption, but dive a little bit quicker if we needed to.” [47]* |
| **2D** | Privacy | *“The ‘Did you take your medicine notification’ is a problem. ‘Did you take your medication?’ Anybody in their right mind is going be, ‘What do you mean you take medication?’ It lets them know your sick. You could be hiding it from your family.” [70]* |
|  |  | *”I have nothing to hide.” [68]* |
| **3A** | Acceptability | *“I found the progress part very useful. I got a reality check when I seen what I was doing and thought I was more active than I am” [36]* |
|  |  | “*He looks so real, and he’s a nice attractive man, and I’m going to ask him a lot of questions about medication! This is genius idea! Thank you! People can go on their phone. People say, I’m afraid to take my meds. This thing can talk back to say, ‘It’s okay.’ Here’s why to take your meds.” [70]* |
| **3B** | Ergonomics | *“I think the app is easy and very clear. And there is not too much in it. With some apps you are like “Where do I find that again?”, but that is not a problem with this one.” [32]* |
| **3D** | Difficulties | *“...that’s too small an interface for my eyes because I’ve had retinopathy, I’ve had laser surgery on both eyes, I’ve had cataracts removed off both eyes.” [31]* |
|  | Favorite things about the app | *“I really like the goals, it motivates you. And it is good that you can change your goal each week, with the 4 options.” [32]* |
|  | Tips for solving problems | *“Well if you have your contact details there that if you are stuck, eh you can ring in.” [36]* |
|  | Comfort | *“I kind of felt like you were entering it in when you did something for the day, then you had to enter it (completed goals) again when you achieved the goal. Can’t they (goals and goal tracker) just be linked? I just feel like they (goals and goal tracker) should link.” [65]* |
|  |  | *“I think the app is easy and very clear. And there is not too much in it. With some apps you are like ‘Where do I find that again?’, but that is not a problem with this one.” [32]* |
| **Session** | **Themes** | **Verbatim lines (only when qualitative tool was applied) from participants (patients, health care professionals or other stakeholders)**  [In brackets number of reference] |
| **4A** | The chance to generate more content | *“If the individual entries can generate a report, that is either printable or emailable to the doctor; that makes sense to me. In other words, it’s creating a history.” [48]* |
|  | Customization | *“Make him functionable like I can brush his hair or take his medicine, drink water, get rest. Have the alarm clock to be aware of his medicine” [70]* |
|  |  | *“The text messages, I thought, were too generic. I thought that they should be specifically more to your accomplishments, and not just the general daily thoughts of the people that are programming it.” [88]* |
| **4B** | Information adequacy | *“I really would like to have [a list that] is relevant to me, so...proven high purine fruit and veg and drinks, yes, but then allow me to create my personal, relevant lists...so as I’m putting in an attack I can access the relevant triggers that have caused me issues in the past.” [48]* |
|  |  | *“It’s so handy especially if you’ve got no idea. In my case I don’t see a lot of the wound so knowing the size is handy because then I can tell whether it’s actually a problem or becoming more of a problem than you know just going along and all of a sudden, and I’ve done it before, going along well and all of a sudden my wound’s fifteen by three or something, which is not ideal.” [31]* |
|  | Utiliy | *“Well if you have your contact details there that if you are stuck, eh you can ring in.” [36]* |
|  |  | *“I think it’ll be useful in my life because...I’ll go to the gym and I have this to do my warm-up...shows me what weights to do, you know,...Because when you go sometimes you just haven’t a clue and you’re kind of doing stuff and you could hurt yourself, you could overdo it, it’s perfect, you know exactly what you’re doing and...keeps you healthy.” [36]* |
|  |  | *“I would love to do this because I know it helps your balance… I think the exercise videos are good, because a lot of the movements are what you do in therapy. So, this is along that line to get you moving more” [49]* |
|  |  | *“There were facts that I didn’t know, which was really cool.” [65]* |
|  |  | *“I was challenging myself to do better and to go farther than previous...I knew what I did the day before and was like, “How do I challenge myself to go a little bit farther, to push myself a little bit hard today*?.” *[88]* |
| **4C** | Hardware Limitations | *“Mine (app) got a bit slow sometimes” [65]* |
|  |  | *“It’s very small and I can’t see what it is.” [58]* |
