# Supplementary material for: Proposal for the User-Centered Design Approach for Health Apps Based on Successful Experiences: Integrative Review
Source: JMIR Mhealth Uhealth. 2020 Apr 22;8(4):e14376. doi: 10.2196/14376 (PMC7203616; doi:10.2196/14376)
Supplement: Multimedia Appendix 3 [file mhealth_v8i4e14376_app3.pdf]

Session 3: Does the app fit the needs of final users?

- a) **Acceptability** [36, 69, 77]
  - Same examples of questions in focus group using likert scale from 1 to 4 [75]
    - *Apps is acceptable to…*
    - *App is helpful to…*
      - *Any kind of functionality that app offers*
    - *App is useful to learn about…*
    - *Reminders in app are useful to…*
      - *Main topic of the app*
    - *Functionalities in app are acceptable to receive self-monitoring information*
  - Service user technology acceptability questionnaire -SUTAQ- [62, 70, 74]
    - *Utility of the kit [app or any technological solution]*
    - *Effects on health status*
    - *Effects on access to care*
    - *Effects on health care / social care*
    - *Privacy*
    - *Suitability of the kit [app or any technological solution]*
    - *Satisfaction with the kit [app or any technological solution]*
- b) **Ergonomics** [29, 32, 49, 59, 60, 77]
  - Heuristic usability [29, 32, 59, 60]
    - *Heuristic 4: Good ergonomic and minimalistic design*
- c) **Glanceability** [18, 29, 32, 59, 60, 78, 79]
  - Heuristic usability [29, 32, 59, 60]
    - *Heuristic 5: Ease of input, screen readability and glanceability*
  - Model from Gouveia et al [80]
    - *Being abstract*
    - *Integrating with activities*
    - *Comparison to targets and norms*
    - *Being actionable*
    - *Leading to checking habits (novelty / scarcity)*
    - *Proxy to further engagement*
- d) **Comfort** [44, 75, 81, 82]
  - Some questions to explore comfort [31-33, 36, 39, 64, 71, 75]
    - *If difficult, did you need help from others in using this new technology?*
    - *Any suggestions to resolve difficulties?*
    - *Any preference features?*
    - *Any privacy concerns?*
    - *What issues or problem did you experienced with the app?*

[In brackets number of reference]
