# Supplementary material for: Proposal for the User-Centered Design Approach for Health Apps Based on Successful Experiences: Integrative Review
Source: JMIR Mhealth Uhealth. 2020 Apr 22;8(4):e14376. doi: 10.2196/14376 (PMC7203616; doi:10.2196/14376)
Supplement: Multimedia Appendix 4 [file mhealth_v8i4e14376_app4.pdf]

Session 4: Last Testing. Keep on Improving

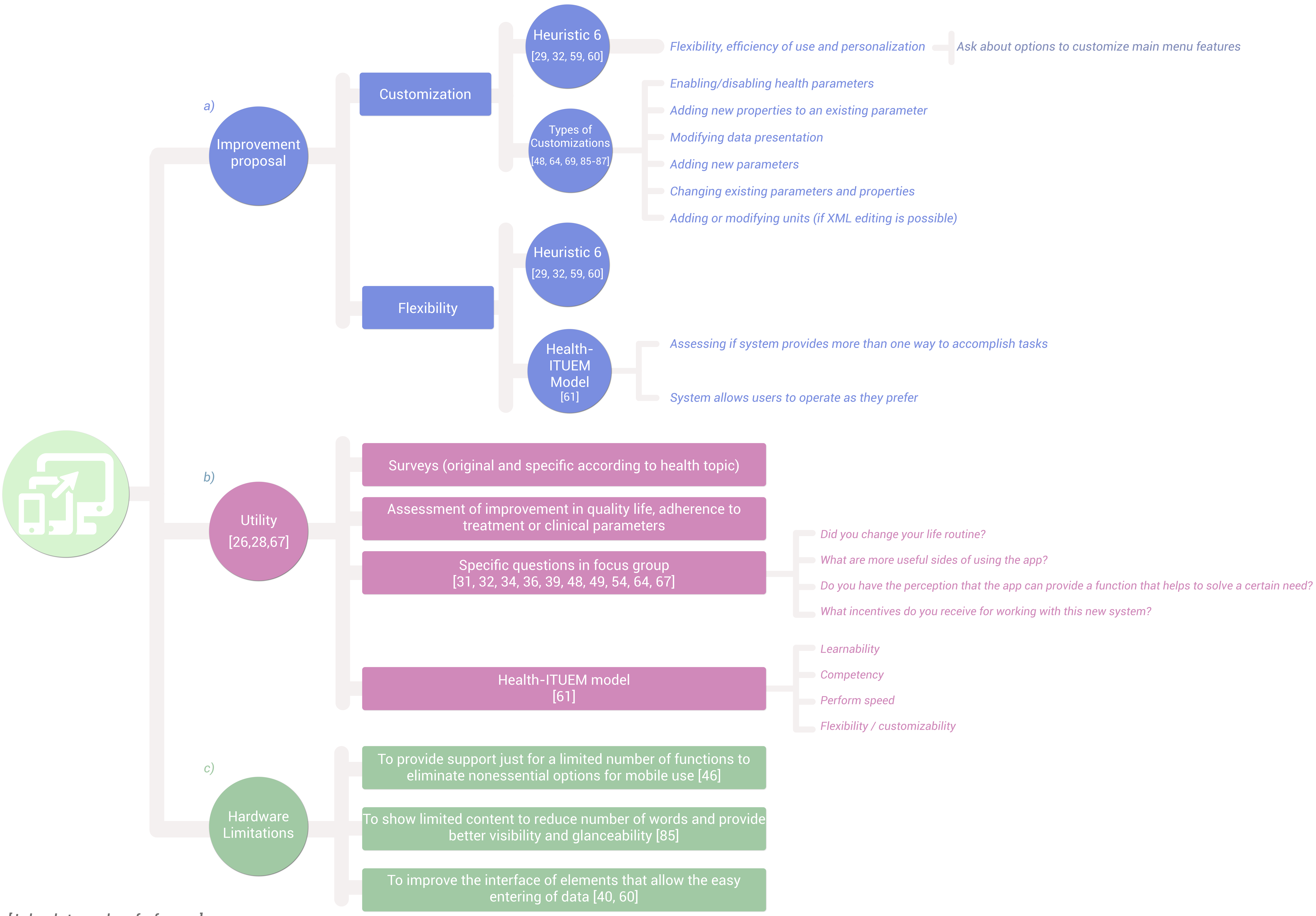

[In brackets number of reference]
